# Zinc Nutritional Status in Patients with Cystic Fibrosis

**DOI:** 10.3390/nu11010150

**Published:** 2019-01-11

**Authors:** Marlene Fabiola Escobedo Monge, Enrique Barrado, Carmen Alonso Vicente, María Paz Redondo del Río, José Manuel Marugán de Miguelsanz

**Affiliations:** 1 Faculty of Medicine, Valladolid University, Avenida Ramón y Cajal, 7, 47005 Valladolid, Spain; pazr@ped.uva.es; 2 Department of Analytical Chemistry, Science Faculty, University of Valladolid, Campus Miguel Delibes, Calle Paseo de Belén, 7, 47011 Valladolid, Spain; ebarrado@qa.uva.es; 3 Department of Paediatrics of the Faculty of Medicine, Valladolid University; Section of Gastroenterology and Pediatric Nutrition, University Clinical Hospital of Valladolid, Avenida Ramón y Cajal, 7, 47005 Valladolid, Spain; carmenalonso@gmail.com (C.A.V.); jmmarugan@telefonica.net (J.M.M.d.M.)

**Keywords:** serum zinc concentration, hypozincemia, dietary zinc intake, dietary zinc deficiency, cystic fibrosis, marginal zinc deficiency

## Abstract

Background: Zinc is an essential nutrient for all forms of life and its deficiency affects the normal growth and development of human beings. Objective: The main aim was to investigate zinc nutritional status by serum zinc concentration (SZC) and dietary zinc intake and their association in cystic fibrosis (CF) patients. Methods: A cross-sectional study was conducted in CF patients. Anthropometric measurements and respiratory and pancreatic tests were conducted. Hypozincemia was determined by SZC while using atomic absorption spectrophotometry and dietary zinc deficiency by prospective 72-h dietary surveys. Results: Mean SZC (87.2 ± 16.7 μg/dL) and dietary zinc intake (97 ± 26.9% Dietary Reference Intake) were normal. Three of 17 patients with CF (17.6%) had hypozincemia and four (23.5%) had a dietary zinc deficiency. No patient with dietary zinc deficiency had hypozincemia. A positive and significant association was observed between SZC and Z-score of BMI-for-age (*p* = 0.048) and weight-for-height (*p* = 0.012) and between dietary zinc intake and energy intake (EI, *p* = 0.036) and Z-score of weight-for-high (*p* = 0.029). Conclusion: SZC was associated with the nutritional status, expressed as BMI (Body Mass Index) and weight-for-height Z score, and dietary zinc intake with EI and weight-for-height Z-score. No patient with hypozincemia had dietary zinc deficiency. This situation should alert us to a marginal zinc deficiency and it may explain why there were no overlapping cases between the two groups. We suggest that probably 41% of the cases in this study would be at elevated risk of zinc deficiency and a zinc supplementation may be considered.

## 1. Introduction

Cystic fibrosis is a chronic autosomal recessive disorder that affects the lungs and the digestive system of approximately 70,000 people worldwide and is caused by mutations in the cystic fibrosis transmembrane conductance regulator (CFTR) gene [1]. Nearly 2000 different disease-causing mutations have been identified, but the most common mutation (approximately 70% of patients) is a three-base-pair deletion (CTT) in exon 10. This mutation causes an in-frame deletion of a phenylalanine residue (DF508) and it results in the lack of a functional cAMP-gated chloride channel on the apical surface of secretory epithelial cells [2]. Thus, CF epithelia fail to transport chlorine and water, and morbidity in CF often results from lung disease [3]. The gastrointestinal manifestations of CF include exocrine pancreatic insufficiency and intestinal mucosal abnormalities [4]. The incidence of CF is about one in 3500 white births in Europe. The mean prevalence in the United States and de European Union (EU) is similar, 0.74 and 0.80 in 10,000 people, respectively [1]. Growth and development in patients with CF can be impacted by several factors that are associated with the disease, such as nutritional status and zinc in particular. 

The solution and complexation chemistry of zinc ions is the basis for zinc biology [5]. Zinc is a ubiquitous trace element [6,7]. Zinc is the 23rd most abundant element in the earth’s crust [8], having atomic number 30 and atomic weight 65.37, is vital in the living world [9]. Zinc is an essential trace element that participates in many metabolic processes as a catalytic, regulatory, and structural component [10]. It is a vital cofactor for the function of more than 10% of proteins that are encoded by the human genome (approximately 3000 proteins/enzymes) [11,12]). Zinc-dependent proteins play numerous indispensable roles within cells, such as transcriptional regulation, DNA repair, apoptosis, metabolic processing, extracellular matrix (ECM) regulation, and antioxidant defence [13]. It is also involved in the metabolic hormone regulation of growth and it has key roles in gene expression regulation and the immune system. Zinc also regulates thousands of genes through the metal response element (MRE) binding transcription factor-1 (MTF-1), and zinc controls numerous cell-signalling pathways by modulating kinase and phosphorylase activities. The coordination of these zinc-dependent cellular functions appears to be related to one or several of the 24 zinc transporters that exhibit differing specificity of expression in different cell types [14,15]. 

Zinc is important for human health and disease due to its critical roles in growth and development [6,7], bone metabolism, the central nervous system, immune function, and wound healing [16]. Not only in physiological but also mental and behavioral terms [17]. Zinc is essential for a wide variety of cellular processes in all cells [18]. Cells with a rapid rate of turnovers, such as those of immune, gastrointestinal systems, and skin, are particularly vulnerable to zinc deficiency, accounting for the initial effects of dermatitis, diarrhoea, alopecia, and loss of appetite [19]. Zinc deficiency impairs both the specific susceptibility to bacterial, viral, and fungal pathogens [20]. This triggers an array of health problems in children, many of which can become chronic and serious, such as weight loss, stunted growth, weakened resistance to infections, and early death [21]. The early effects of zinc deficiency are chemical, functional, and may be “hidden”. Some examples of functions at risk are: gene expression, fetal growth and development, child and adolescent growth, wound healing, immunity, resistance to oxidative stress and inflammation, brain development, and neuropsychological function [22]. 

Adequate zinc nutriture is critically important for human health [11]. Inadequate dietary intake of absorbable zinc is one of the major causes of zinc deficiency [23]. Zinc is a critical dietary nutrient, particularly in the early stages of life; epidemiological data suggest at least one in five humans is at risk of zinc deficiency [18]. It is present at less than 50 mg/kg in the human body [16]. At 2–3 g in total, zinc is the second most abundant metal in humans and it is distributed unequally throughout different organs and tissues. Prostate, pancreas, and bone are considerably high in zinc, containing up to 200 µg/g. In contrast, zinc concentrations in the heart, brain, and plasma are comparatively low, at 1–23 µg/g. Although plasma has only 1 µg/g, it is probably the most important reservoir for zinc homeostasis [24]. Zinc storage capacity seems to be highly vulnerable to reduced dietary intake or increased losses due to illness [25]. The concept of body zinc status is based on the notion of acquisition of zinc that is sufficient for optimal biological processes. This may be a consequence of dietary zinc intake, phytate consumption, gastrointestinal health, rate of zinc excretion and reabsorption, and other factors, many of which are not clear. 

Zinc deficiency can be both inherited and acquired [26,27]. Although severe zinc deficiency is extremely uncommon in European populations, marginal deficiency may be much more prevalent and is associated with immune system dysfunction and restricted physical development [28]. Marginal zinc deficiency is characterized by slight weight loss, rough skin, oligospermia, and hyperammonemia [24,29]. The clinical signs of marginal zinc deficiency include a decrease in immunity, taste and smell senses, night blindness, memory compromise, and decreased spermatogenesis [29]. Clinical diagnosis of marginal zinc deficiency in humans remains problematic. Acquired deficiency of zinc has now been reported in many diseases, such as cirrhosis of the liver, chronic renal disease, malabsorption syndrome, chronic alcoholism, sickle cell disease, and other chronic diseases, including malignancies [30]. WHO (World Health Organization)/UNICEF (United Nations International Children’s Emergency Fund)/IAEA (International Atomic Energy Agency)/IZiNCG (International Zinc Nutrition Consultative Group) bodies recommend the use of three indicators to value zinc status at the population level: prevalence of intakes below the estimated average requirement, percentage of low serum zinc concentration, and percentage of children less than five years who are stunted [31,32]. There are four main intervention strategies for combating zinc deficiency, which include dietary modification/diversification, supplementation, fortification, and bio-fortification [11]. 

The overall goal in CF is that every patient should achieve normal growth. In CF patients, zinc deficiency can be common due to disturbed protein intake and fat malabsorption [33]. Zinc deficiency can lead to a broad range of consequences [34] and symptoms, such as stunted growth, delayed sexual maturation, disturbed immunity, poor appetite, and diarrhoea, each of which is frequently present in patients with CF [35]. Young infants, because of their rapid growth rate, have relatively high physiologic zinc requirements [36]. Approximately 35% of the children with CF aged between five and 10 years were either nutritionally ‘at risk’ or in ’urgent need’ of nutritional intervention and rehabilitation. CF patients are at risk of zinc deficiency, which is a manifestation of steatorrhea [37]. Damphousee et al. point out that almost a quarter of adults with cystic fibrosis who had a good nutritional status had a low plasma zinc concentration, and those with a low plasma zinc concentration had a poor clinical outcome [38]. 

Zinc is involved in many metabolic and chronic diseases, such as: diabetes, cancer (oesophageal, oral small cell carcinoma, breast cancer), and neurodegenerative diseases. There is also strong evidence between zinc deficiency and several infectious diseases, such as malaria, HIV (human immunodeficiency virus), tuberculosis, measles, and pneumonia [39]. At the moment, the real extent of the prevalence of zinc deficiency in the world is unknown, and we have nothing more than a very rough estimate not only of the prevalence, but also of the potential impact on global health that can be expected from improving zinc nutrition in populations [40]. The partial ignorance of zinc deficiency as a public health problem makes it necessary to carry out studies to know more about zinc nutritional status in CF patients. Therefore, the main aim of the study was to evaluate zinc status in CF patients by assessing their SZC and dietary zinc intake. 

## 2. Materials and Methods

A cross-sectional study was conducted including both paediatric and adult CF patients with homozygous Delta F580 and other related mutations, which were consecutively referred for nutritional assessment to the Paediatrics service at the University Clinical Hospital of Valladolid, in a period of 18 months. The local ethics committee at the University Clinical Hospital reviewed and approved this study. Anthropometric assessment (weight, height) using standard techniques and Z-score of weight-for-age, height-for-age, weight-for-height, and BMI were calculated using Orbegozo Tables [41]. The growth rate of CF patients was calculated by using clinical history data. The Score Norman-Crispin (>5), forced vital capacity (FVC% < 80%), and forced expired volume in 1 s (FEV_1_ < 80%) predicted value by spirometry to estimate respiratory-insufficient (RI), and the coefficient fat-absorption (CFA > 94%) by 72-h quantitative faecal fat collection to value pancreatic-sufficient (PS) and insufficient (PI) were realized. All patients were treated with pancreatic enzyme replacement therapy (PERT) and fat-soluble vitamin supplements (Table 1). 

Fasting blood samples were collected and all research procedures minimized the risk of contamination. The SZC was conducted at Chemistry Department of the University of Valladolid using an atomic absorption spectrophotometer (model PU9400 Philips) [42]. Serum zinc <70 μg/dL in children <10 years of both sexes and in women aged ≥10 and <74 μg/dL in men aged ≥10 were used as cutoff to evaluate hypozincemia [31]**.** We examined the activity of acute phase proteins as inflammatory markers, including C-reactive protein (CRP) > 4 U/L and erythrocyte sedimentation rate (ESR) woman > 20 mm/h, men > 15 mm/h, by standardized methods. Diet analysis was made through a prospective dietary survey of 72 h (including one of the weekend days). From that, daily EI (calories) and dietary zinc intake based on estimated portions and referred for the percentage of Dietary Reference Intake (%DRI) was assessed using the Mataix Food and Health computer program [43]. In order to assess inadequate dietary zinc intake, we used the cut point <80%DRI. 

**Figure 1:**
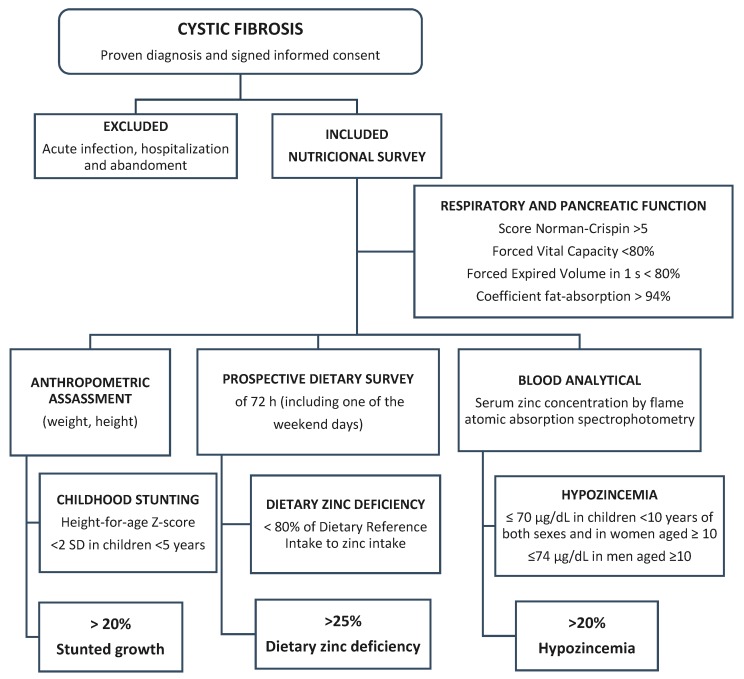
Graphic of design of the cross-sectional study to value the risk of zinc deficiency.

The marginal or subclinical zinc status is defined as a deficit state without pathognomonic clinical signs and with an acceptable (normal values) SZC in patients with dietary zinc deficiency [44]. The risk of zinc deficiency by height-for-age of growing infants and children (>20% of stunting), dietary zinc intake (>25% of dietary zinc deficiency), and SZC (>20% of hypozincemia) was used to estimate zinc status (Figure 1). Data are expressed as mean ± standard deviation score (SDS), median, ranges, and percentages. Differences between normal and deficient groups with nutritional status variables were assessed using unpaired Student *t*-tests or Wilcoxon rank tests for non-normally distributed variables. Pearson correlation coefficients were performed to test for significant associations among the same variables. The significance level was set at *p* < 0.05. Analyses were performed using the SPSS/PC software (IBM Corp., Armonk, NY, USA).

## 3. Results

In this cross-sectional study, seventeen CF patients (10 females, seven males) were studied, and their characteristics are summarized in Table 1. The mean age was 14.8 ± 8 years with median 15 and range was 2–31 years (seven children, five adolescents and adults). Seven CF patients have homozygous Delta F580, and other six patients have other CFTR mutations. The genotype of cystic fibrosis subjects was elaborated by Biological and Molecular Genetics of the School of Medicine at Valladolid University (Table 2). The 29.4% (5/17 cases) were malnourished (BMI < −2SDS). The 64.7% of CF patients had RI (mean Score Norman-Crispin 6.3 ± 5.5 and FEV_1_ 77.7 ± 26.1%) and 94.1% PI (mean coefficient fat-absorption 88.4 ± 8.6%). The nitrogen balance was positive but lower (mean was 3.7 ± 5.3). There was no significant difference in weight-for-age, height-for-age, weight-for-height, and BMI Z-score, according to gender, pancreatic, and pulmonary function. However, PS patients had less BMI than PI patients (*p* = 0.020). No patients had stunted growth. The growth rate in patients with hypozincemia was normal; only one patient with IP had a growth rate that slightly decreased. The median FEV_1_ was 78.9% and FVC% was 78.8%. There were 17.6% of patients colonized with *Pseudomonas aeruginosa*, *Candida spp*, and 23.5% by *Staphylococcus aureus*. Their lung function was not a significantly worse than those without such colonisation (Table 3). There were 13 patients (76.5%) with steatorrhea and nine (52.9%) with respiratory insufficiency. Weight-for-age was not affected by IP and was normal in patients with zinc deficiency intake. 

**Figure 2:**
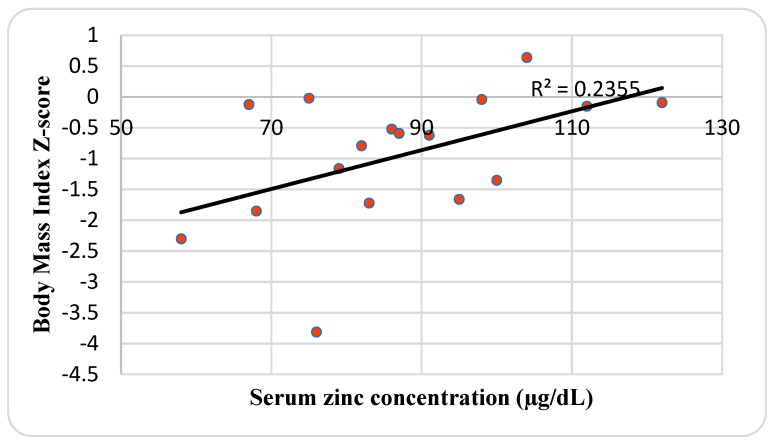
Relationship between serum zinc concentration and Body Mass Index Z-score.

**Figure 3:**
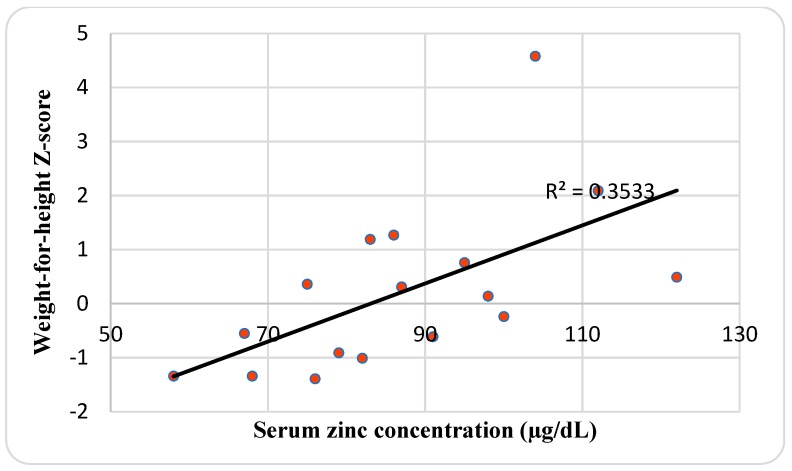
Relationship between serum zinc concentration and weight-for-height Z-score.

**Figure 4:**
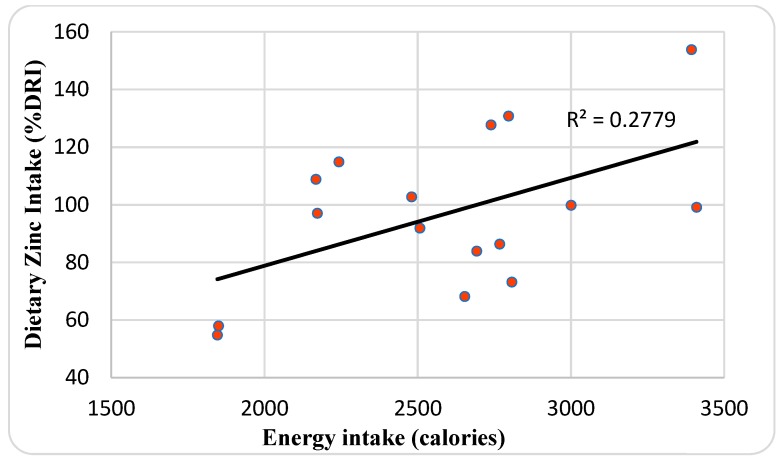
Relationship between dietary zinc intake and energy intake. %DRI: Percentage of Dietary Reference Intake.

**Figure 5:**
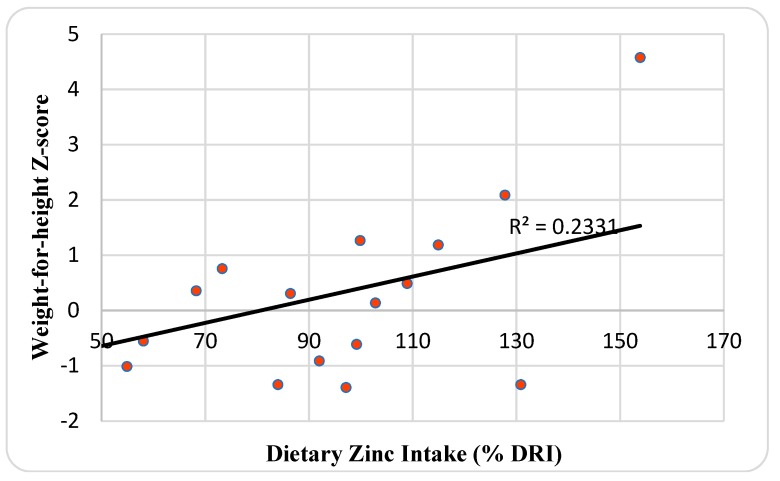
Relationship between dietary zinc intake and weight-for-height Z-score. %DRI: Percentage of Dietary Reference Intake.

The mean SZC of 87.2 ± 17 μg/dL and it ranged between 58 and 122 μg/dL. Although males had a higher SZC (93.3 μg/dL) than females (83 μg/dL), it was not significant. The mean BMI and weight-for-height Z-score SDS was −0.95 ± 1.1 and 0.22 ± 1.51, respectively. There was a positive correlation between SZC and BMI Z-score (*r* = 0.49; *p* = 0.048) (Figure 2), and weight-for-height Z-score (*r* = 0.59; *p* = 0.012) (Figure 3). CRP and ESR levels were normal except in three (17.7%) and two patients (11.9%), respectively. ERS and CRP were slightly increased in a single patient with hypozincemia (23 mm/h) and zinc intake deficiency (6.9 U/L), respectively. The mean of dietary zinc intake was 97 ± 26.9%DRI (14.5 ± 4 mg/day) and it ranged between 54.9 and 153.9% (8.2–23.1 mg/day), and EI was 2594.9 ± 464.5 and ranged between 1846 and 3409.6 kcal. Females had a higher dietary zinc intake (105.8%) than males (85.6%), although males (2631.1 Kcal) had an insignificantly higher than females (2566.9 Kcal). There was a positive correlation between dietary zinc intake and EI (*r* = 0.53; *p* = 0.036) (Figure 4), and weight-for-height Z-score (*r* = 0.55; *p* = 0.029) (Figure 5). There was no significant difference in SZC by respiratory and pancreatic function. Conversely, patients with RI had dietary zinc intake that was significant higher than normal patients (*t =* 0.015) (Table 3).

The analysis showed that there were 17.6% of CF patients with hypozincemia, 23.5% with dietary zinc deficiency, and no patients under five years old had growth stunted. Three women—one infant eutrophic DF508/2183AA-G, one adolescent, and adult malnourished DF508 homozygous—had hypozincemia. Four patients had a dietary zinc deficiency, three-male—one infant eutrophic DF508 with a mutation in intron 12 (1898 + 1G → A), one adolescent malnourished DF508/1717-1G, and one adult eutrophic DF508/S549R—and one-woman adult eutrophic DF508 homozygous. No patient with dietary zinc deficiency had hypozincemia.

## 4. Discussion

There is a strong plausibility for zinc deficiency in infants and children with CF. Zinc deficiency has been documented in young infants identified by newborn screening prior to the initiation of pancreatic enzyme therapy [4]. In settings without newborn screening, the presentation is typically later in infancy, with associated growth faltering, diarrhoea, and dermatitis similar to acrodermatitis enteropathica [45]. Investigators have examined the relationship between zinc, body composition, growth, and pulmonary function in older children and adults with CF after the original report by Halsted and Smith in 1970 [46]. Nowadays, the use of zinc-finger nucleases to permanently and precisely modify the human genome offers a potential alternative to cDNA-based gene therapy in subjects with CF [2]. In Spain and in the EU there are few studies of zinc nutritional status in CF patients. Therefore, the main aim of the present study was to evaluate zinc nutritional status in CF patients by determining the percentage of cases with hypozincemia, stunted growth in children less than five years and inadequate dietary zinc intake. 

Zinc, an almost omnipresent metal ion, is not only a vital element in various physiological processes but also a drug in the prevention and management of many diseases. Nearly half of the world’s population is at risk for inadequate zinc intake, suggesting that public health programs are urgently needed to control zinc deficiency. The inability to link the physiological effects of zinc depletion to zinc status is due, in part, to an incomplete understanding of the biochemical and physiologic functions of zinc [10]. Zinc’s anticopper action is unique [47]. A moderate level of zinc deficiency has been observed in many gastrointestinal disorders. These include malabsorption syndrome, Crohn’s disease, regional ileitis, and steatorrhea. In 1968, MacMahon et al. [48] were the first to report zinc deficiency in a patient who had steatorrhea. Zinc deficiency in patients with malabsorption syndrome is now well recognized, and most physicians are aware of this problem [49]. 

The lack of generally accepted biomarkers of zinc status has further impeded the estimation of the global prevalence of zinc deficiency [47]. No single body zinc compartment represents an adequate estimate of overall body zinc status [31,32]. The normal concentration of zinc in human blood serum and urine (24 h) is 800 ± 200, 109 to 130, and <500 μg/dL, respectively. The mean serum zinc concentration is 1 mg/L [50]. Red blood cells contain about 10 times higher concentration than that in the serum. Whole blood has about five times the serum concentration [51]. A thorough assessment of zinc status by current methods is complex. For now, the simplest measure is plasma zinc, which is 71% sensitive for clinically apparent deficiency [25], at a cutoff of 40 mg/dL [52]. It is insensitive for marginal (subclinical) deficiency. Thus, it is difficult to identify zinc deficiency in association with acute diarrhoea and to know how to monitor efficacy of replacement in terms of zinc nutrition [7]. 

A sensitive, specific biomarker of zinc nutrition has not been identified for the individual diagnosis of zinc deficiency. The most commonly used measurement of zinc status is plasma or serum zinc, despite the fact that less than 0.1% of body zinc is present in plasma and the concentration appears to be under strict homeostatic control. Despite these limitations, the fact that plasma zinc concentrations are normally distributed in healthy populations makes it possible to establish reference values to be used to identify individuals at risk for a low zinc status [53]. Therefore, plasma or serum zinc concentration is the only biochemical indicator to assess the zinc status of populations recommended by WHO/UNICEF/IAEA/IZiNCG [31].

In spite of the fact that the number of CF patients in the study is small, we must take into account that the results show that median SZC (86 μg/dL) in this series was normal and 17.6% of patients had hypozincemia. The median did not differ significantly from the National Health and Nutrition Examination Survey (NHANES) II study (88 μg/dL) [31]. In a study of 30 children with CF (mean age 8.65 ± 3.01 years) in a Children’s Medical Centre in Tehran, the mean SZC was 111.43 µg/dL, which is significantly higher than results in age-matched controls (91.43 µg/dL, *p* < 0.00) [33]. Ataee et al. found that CF patients had 29.4% malnourished, 94.1% PI, and 64.7% RI [33]. This is in contrast to the present study, where only two women (11%) with hypozincemia—one adolescent and adult—were malnourished (mean BMI − 2SDS), and the adolescent had PI (fat absorption coefficient 91.74%) and RI (FEV_1_ 52%). Maqbool et al. proved an association between suboptimal plasma zinc, poor nutrition, and growth status in infants with CF and PI. Growth faltering observed in older children with CF is undoubtedly multifactorial, including malnutrition, however, in one report older children’s total dietary zinc intake and zinc status were adequate and unrelated to growth status [54]. 

Borowitz et al. point out that hypozincemia in CF is difficult to characterize, because zinc deficiency may be present when plasma zinc is in the normal range [55]. In a study, increased zinc loss in 101 patients (mean age 16 years) with persisting steatorrhea was found, and 12.6% of this population had SZC below the *p*-value of 2.5 of the NHANES II, so there could have an increased risk of zinc deficiency in some CF patients [56]. Although hypozincemia has been reported in approximately 30% of young infants with CF in newborns screenings [33], Maqbool et al. reveal that prevalence of low plasma zinc ranging from 0% to 40% in various populations of infants, children, and adolescents with CF [54]. There have been several reports of young infants with CF presenting with signs and symptoms of severe zinc deficiency [36], such as growth retardation, increased susceptibility to infections, delayed sexual maturation, eye problems, and anorexia that are caused by a reduced sense of taste (hypogeusia) [1]. 

In this study, no patient had growth stunted. The growth rate in these patients with CF and hypozincemia was normal. Only one patient with IP had a growth rate slightly decreased. Up to now, no studies have been able to demonstrate a relation between serum zinc and growth parameters. However, the decrease in insulin-like growth factor 1 (IGF-1) concentrations correlate with the nutritional status of CF patients. Zinc supplements could theoretically have beneficial effects on energy expenditure, appetite, nutritional status, as well as growth. Only one retrospective study examined and demonstrated an improved EI during zinc supplementation in CF patients with low serum zinc. CF patients may theoretically benefit from zinc supplements in several different aspects of their disease. A retrospective analysis of clinical data suggests improved appetite, EI, nutritional status, as well as pulmonary function with zinc supplements [37]. Van Biervliet et al. show that high-dose (5 mg zinc/kg/day) supplementation was associated with beneficial effects on growth and pulmonary function [57]. However, no controlled trials are available that replicate these effects. 

However, many studies have shown that zinc plays a role in promoting physical growth and the development of intelligence in children [58]. Young children are at greater risk of zinc deficiency because of increased zinc requirements during growth. Exclusively breastfed infants of mothers with adequate zinc nutriture obtain sufficient zinc for the first 5–6 months of their life [59]. Children are particularly sensitive to a suboptimal state of zinc during periods of rapid growth that create a higher need for zinc that may not be met [60]. It seems to be that zinc is the most important factor in the needs related to the deposit of new tissue, since 20 mg of zinc are needed for each kg of muscle mass [61]. Therefore, a higher growth rate corresponds to higher net zinc retention [62]. After this age, complementary foods containing absorbable zinc are required to satisfy their requirements. The physiological requirements for zinc peak during adolescence at the time of the pubertal growth spurt, which generally occurs in girls between 10 years and 15 years and in boys between 12 years and 15 years. Even after the growth spurt has ceased, adolescents may require additional zinc to replenish depleted tissue zinc pools [63]. On the other hand, a marginal zinc status during the outbreak of pubertal growth has been associated with slow skeletal growth, maturation, and reduction in bone mineralization [31]. 

Recent studies have demonstrated that a well-balanced diet leads to an improved redox status, which positively affects reducing the risk of non-communicable chronic diseases [64]. An inadequate intake of micronutrients at any stage of life affects various functions within the immune system, manifesting in decreased resistance to infections and an increase in the severity of symptoms. For example, zinc deficiency can increase thymic atrophy, decrease lymphocyte number and activity, and increase oxidative stress and inflammation by altering cytokine production [65,66]. As a result, the risk of all types of infection (bacterial, viral, and fungal), but especially diarrhoea and pneumonia, is increased [67]. 

In this study, the median dietary zinc intake for CF patients (98%DRI) was normal and 23.5% of them had dietary zinc deficiency with less than 80%DRI and approximately 5.9% exceeding the upper level (UL). Median dietary zinc intake in this study was significantly lower than the NHANES II control group (178% Recommended Dietary Allowance) (*p* < 0.00) [68], and did not differ significantly from the ANIBES study (83%). ANIBES (Anthropometry, Intake, and Energy Balance in Spain) was a Spanish study that evaluates energy intake and expenditure, body composition, and dietary patterns in a national representative sample of subjects. The subjects that were excluded from the study were those that were on a prescribed diet due to medical tests, pre- or post-surgery situation, diagnosed disease or any pathological or physiological situation, or those with any disease or illness (e.g., cold, gastroenteritis, chicken pox, etc.). Here, Olza et al. show that the percentage of the Spanish population included in the ANIBES study, as in this study, not meeting the European recommendations for zinc. Even when the plausible energy reporters were analysed separately, these percentages remained above 40% [69].

In spite of the fact that 23.5% of patients had inadequate dietary zinc intake, none of them had hypozincemia (17.6%). Low serum zinc concentrations can occur in the presence of several conditions, representing a normal physiologic response and they are not necessarily indicative of low zinc status [11]. Zinc can be temporarily redistributed from plasma to other tissue or the concentrations can be changed by conditions that are unrelated to zinc status. Infections, fever, food intake, and pregnancy lower plasma zinc, whereas starvation and catabolism increase it. Plasma zinc is also depressed at times of rapid tissue growth [70]. Cellular, tissue, and whole-body zinc homeostasis are tightly controlled to sustain metabolic functions over a wide range of zinc intakes, making it difficult to assess zinc insufficiency or excess [71]. In many moderate zinc dietary restrictions (3–5 mg/day), the response is more inconsistent, with a slight decrease or no change in serum zinc concentrations, possibly related to the duration of the dietary restriction and the relationship phytate:zinc molar ratio from the diet. Serum zinc levels fall clearly when the dietary zinc intake is less than approximately 2 to 3 mg, reaching a plateau when zinc intake reaches approximately 25 to 30 mg/day [31]. This means that, in populations with a high prevalence of inadequate zinc intake, there would be a higher prevalence of low serum zinc concentration [72]. Thus, the prevalence of low concentrations of serum zinc may be indicative of the risk of zinc deficiency in the population [31,40]. It is striking that, in spite of this fact, no more cases of hypozincemia will be presented in this study. 

A retrospective study in 304 adults CF patients with good nutritional status and moderate lung disease reported that one-fifth of them (22.4%) were deficient in zinc (<60 µg/dL), regardless of their nutritional status, as BMI was not found to differ significantly between zinc deficient and sufficient patients [38]. This is in contrast to the present study, where SZC was associated with nutritional status, expressed as BMI Z-score (*p* = 0.048) and weight-for-height Z-score (*p* = 0.012), and dietary zinc intake with EI (*p* = 0.036) and weight-for-height Z-score (*p* = 0.029). These data suggest that zinc is likely to be a growth-limiting factor in this group of CF patients, which is mainly due to an acquired deficiency of zinc secondary to its inherited chronic disease, which increases the losses of zinc by steatorrhea. There was no significant difference in anthropometric, as assessed according to gender, pancreatic, and pulmonary function, except for lower BMI in patients with PS than PI patients (*p* = 0.002). 

Those observations are not surprising, because the gastrointestinal manifestations of this common heritable condition include exocrine pancreatic insufficiency and intestinal mucosal abnormalities [54], which produces malabsorption of essential fatty acids, fat-soluble vitamins, and several micronutrients, including zinc [73]. Increasing evidence demonstrates the importance of zinc in CF-affected tissues including the lungs and pancreas [38]. Zinc is particularly abundant in the pancreas, where it is involved in the control of glucagon secretion and digestive enzyme activity. Zinc is very important in the synthesis, storage, and secretion of insulin, as well as in maintaining conformational integrity of insulin in the hexameric form. Any decrease in zinc levels can affect the ability of the islet of Langerhans cell to produce and secrete insulin, and compound the problem, particularly in type II diabetes [74]. Therefore, zinc deficiency is associated with decreased insulin secretion and sensitivity, features that are characteristic of CF-related diabetes [38]. 

It should be understood that the major organ system of zinc exchange with the environment is the gut [25,36]. The current understanding of zinc homeostasis indicates that the primary determinants of zinc absorption are the amount of zinc ingested and dietary phytate, with the latter having a major effect on zinc bioavailability. However, in normal as well as in many pathologic conditions, the gastrointestinal tract is the major site of zinc losses resulting from the secretion of endogenous zinc into the lumen and subsequent excretion in the faeces [44]. Zinc is primarily absorbed by a saturable process in the proximal small bowel but also more distally through entero-enteric reabsorption that diminishes losses [75]. Two generalized dietary patterns are major factors in the aetiology of dietary zinc deficiency; one in which the inhibition of absorption predominates and bioavailability is the issue and the other in which the zinc content of the diet is deficient [76]. The adjustments in gastrointestinal zinc absorption and endogenous excretion are synergistic. Shifts in the endogenous excretion appear to occur quickly with changes in intake just above or below optimal intake, while the absorption of zinc responds more slowly, but it has the capacity to cope with large fluctuations in intake [77]. With extremely low zinc intakes or with prolonged marginal intakes, secondary homeostatic adjustments may augment the gastrointestinal changes. These secondary adjustments include changes in urinary zinc excretion, a shift in plasma zinc turnover rates, and possibly, an avid retention of zinc released from selected tissues, such as bone, in other tissues to maintain function [78,79].

The results showed that some participants still had residual steatorrhea (76.5%), even though all patients except one of them received adequate doses of PERT and fat-soluble vitamins supplements with their diet. The correlation between dietary zinc intake and weight-for-height Z-score was not influenced in the absence of residual steatorrhea. Therefore, patients without PI had a normal weight-for-age Z-score, and there was no correlation significant between them and dietary zinc intake. On the contrary, weight-for-height Z-score had a positive and significant correlation with dietary zinc intake (*r* = 0.58, *p* = 0.038) in patients with PI. Weight-for-age was not affected by IP and it was normal in patients with zinc deficiency intake. 

Pancreatic insufficiency is frequently present in CF and it causes a major problem for zinc absorption. The fact that zinc deficiency increases the susceptibility to childhood diarrhoea while increased losses of endogenous zinc that are associated with diarrhoea further deplete body zinc, resulting in a vicious cycle that merits further study [80]. Recent stable isotope studies have reported increased endogenous faecal zinc losses and decreased zinc absorption in children and infants with CF [44,55]. Untreated pancreatic insufficiency also increases zinc losses. The fractional zinc absorption is improved by PERT. However, some CF patients continue to have steatorrhea despite correct PERT, and might, therefore, be at risk for developing zinc deficiency. Many CF patients have a suboptimal nutritional status despite the nutritional advice and PERT [37]. For infants and children <2 years of age who are not growing despite apparently adequate nutritional intake, a six-month trial of zinc supplementation (1 mg zinc/kg/day) has been recommended [55]. 

Despite treatment with supra-physiological doses of pancreatic enzyme supplements, residual steatorrhoea is a common problem in patients with cystic fibrosis (CF) and pancreatic insufficiency. Strategies to enhance the activity of pancreatic enzymes include decreasing duodenal acidity. Proesmans and Deboec evaluated the effect of omeprazole (Losec), a proton-pump inhibitor, on fat absorption in 15 CF patients (three girls and 12 boys) with confirmed residual steatorrhoea, despite a high dose pancreatic enzyme supplements (Lipase > or =10,000 UI/kg/day). During treatment with omeprazole, median faecal fat loss (g fat/day) decreased from 13 g (quartiles 11.5–16.5 g/day) to 5.5 g (quartiles 4.9–8.1 g/day) (*p* < 0.01). The same improvement was noted when fat absorption was calculated: 87% (quartiles 81–89%) without versus 94% (quartiles 90–96%) with omeprazole (*p* < 0.001). They concluded that omeprazole improves fat digestion and absorption in CF patients with residual faecal fat loss despite maximal pancreatic enzyme substitution [81]. This improvement could contribute to an increase in zinc absorption. However, suppression of gastric acid secretion by omeprazole reduces intestinal absorption of zinc in other studies [82,83]. Farrel et al. point out that PPI use dramatically reduces supplemental zinc uptake and it can result in decreased zinc body stores. Certain individuals on long-term PPI therapy, such as infants being treated for colic, may be at risk for decreased systemic levels of trace metals that are needed for developmental, regenerative, and immunological requirements [83].

The participants were clinically stable at the time of the study. ERS and CRP were slightly increased in a single patient with hypozincemia (23 mm/h) and zinc intake deficiency (6.9 U/L), respectively. C-reactive protein (CRP) is an acute-phase protein that serves as an early marker of inflammation or infection. The protein is synthesized in the liver and it is normally found at concentrations of less than 10 mg/L in the blood. During infectious or inflammatory disease states, CRP levels rise rapidly within the first 6 to 8 h and peak at levels of up to 350–400 mg/L after 48 h. CRP binds to phosphocholine expressed on the surface of damaged cells, as well as to polysaccharides and peptosaccharides that are present on bacteria, parasites, and fungi. This binding activates the classical complement cascade of the immune system and modulates the activity of phagocytic cells, supporting the role of CRP in the opsonization (i.e., the process by which a pathogen is marked for ingestion and destruction by a phagocyte) of infectious agents and dead or dying cells. When the inflammation or tissue destruction is resolved, the CRP levels fall, making it a useful marker for monitoring disease activity [84]. Elevated concentrations of C-reactive protein or other markers of the acute phase response can be used to indicate the presence of infection and should be considered in the interpretation of results [85].

Serum zinc concentrations are reduced during acute infections and inflammation [86]. Corbo et al. point out that acute stress and inflammation may contribute to zinc redistribution, leading to lower plasma zinc concentrations without affecting total body store [87]. Conversely, normal serum levels do not necessarily translate to a sufficient body store of zinc. Zinc deficiency causes thymic atrophy, lymphopenia, and compromised cell- and antibody-mediated responses that increase the rates and duration of infections. Zinc deficiency is characterized by decreased lymphocyte number and function, particularly T cells, increased thymic atrophy, altered cytokine production that contributes to oxidative stress and inflammation [65]; increased risk of bacterial, viral, and fungal infections (particularly diarrhea and pneumonia) [88] and diarrheal and respiratory morbidity [66,89]. Contrariwise, its supplementation produces the restoration of thymulin activity, increased numbers of cytotoxic T cells, reduced numbers of activated T helper cells (which can contribute to autoimmunity), increased natural killer cell cytotoxicity, and reduced incidence of infections [65]. In children, there is a reduction in the duration of diarrhoea and incidence of pneumonia in at-risk children >6 month, but not in children 2–6 month [88]; reduced duration and severity of common cold symptoms; and, improved outcomes in pneumonia, malaria, and diarrheal symptoms [67,90].

In the airway epithelium, zinc is important for ciliary function, wound healing, and suppression of oxidative stress and apoptosis; all of which have been shown to be impaired in CF. Zinc is known to play a regulatory role in the immune system where correlations have been found between plasma zinc and IL-2 levels, natural killer cell activity, and active thymulin in CF children. Zinc status also affects the expression of proinflammatory cytokines, such as TNFα, IL-1β, and IL-8 [38]. Zinc deficiency leads to an increase in oxidative damage in the airways by causing infiltration of inflammatory cells and increases the production of nitric oxide and superoxide. When this deficiency occurs in conjunction with acute damage to the lung or asthma, greater inflammation occurs, increasing the frequencies of hospitalizations [73]. According to Ataee et al., the number of hospitalizations was significantly reduced after zinc administration, which could be due to the preventive effect of zinc on pneumonia, which is a major cause of hospitalization in these patients {33]. Zinc, an acute phase reactant, would shorten the duration of severe pneumonia and time in the hospital. Adjuvant treatment with 20 mg zinc per day accelerated recovery from severe pneumonia in children and could help to reduce antimicrobial resistance by decreasing multiple antibiotic exposures and lessen complications and deaths where second-line drugs are unavailable [91].

Even though data on zinc supplementation among children with cystic fibrosis are limited [33,57,73,92,93], the ESPEN-ESPGHAN-ECFS guidelines on nutrition care for infants, children, and adult with CF suggest zinc supplementation for people who are at risk of zinc insufficiency [1]. At present, despite contradictory findings, many researchers believe that the intake of 30 mg/day of zinc reduces the number of days of antibiotics used to treat respiratory tract infections in these children [73]. The supplementation trial in 13 Swedish children (2–19 years) for six months demonstrated the transient normalization of plasma zinc levels, where all subjects had had low plasma zinc levels at baseline, but growth velocity or lung function did not improve with the normalization of plasma zinc levels [93]. However, Borowitz et al. reported that empiric zinc supplementation as a treatment trial for a period of six months can be considered for CF patients who are failing to thrive or that have short stature [55]. 

In spite of the fact that Sharma et al. in a double-blind randomized placebo-controlled, the study of zinc supplementation in 37 children with CF (age 5–15 y) did not find any significant difference in the need for antibiotics, pulmonary function tests, hospitalization, colonization with pseudomonas, or the need for antibiotics. They highlighted that the majority of the children had low serum zinc levels at baseline; therefore, the dose of zinc administered may not have been sufficient to have an impact on the outcomes [92]. Conversely, Abdulhamid et al. conducted a double-blind randomized controlled small trial in which 26 children were treated with placebo or zinc (30 mg/day) for one year. They documented that the requirement for antibiotics was significantly lower among those receiving zinc than those receiving placebo (*p* = 0.05). The improvement was more pronounced among those who had low plasma zinc at baseline (*p* = 0.02). Reduced levels of plasma interleukin-6 and interleukin-8 were also documented. Furthermore, they recommended that higher daily doses of zinc might be necessary to decrease respiratory tract infections and modify the immune response [73]. 

WHO/UNICEF/IAEA/IZiNCG recommends that if more than 20% of the population (or population sub-group) has SZC below the relevant cut-off, more than 25% has inadequate dietary zinc intake below the EAR and more than 20% of children less than five years old who are stunted, the whole population (or sub-group) should be considered to be at risk of zinc deficiency and of public health concern, and an intervention to improve population zinc status is recommended [32,78]. At that point, in the present study, we have considered four highlights. Firstly, the median SZC was normal (88 µg/dL) [31] but the median dietary zinc intake (98%DRI) was lower from European recommendations for zinc and presented a significant difference from the NHANES II control group (*p* < 0.00) [68]. Secondly, no patient less than five years had stunted growth. Thirdly, the patients with hypozincemia (zone of risk > 20%) and dietary zinc deficiency (zone of risk > 25%) were close to the zone of high risk of zinc deficiency [32,78]. Finally, in spite of the fact that 23.5% of patients had inadequate dietary zinc intake, none of them had hypozincemia (17.6%). 

Taking all highlights into account, we should indicate that this situation of patients with inadequate dietary zinc without hypozincemia should alert us to a state of marginal zinc deficiency, which may explain why there were no overlapping cases between the two groups. Consequently, we suggest that approximately 41% (7/17 cases) of patients with CF (patients with serum zinc deficiency and dietary zinc intake) would be at high risk of zinc deficiency, and that an intervention strategy—dietary modification/diversification, supplementation, fortification, and bio-fortification—should be carried out about these patients with CF. 

One limitation of the study is its small sample size, while its strengths include the determination of the nutritional status of zinc by the percentage of cases with hypozincemia, stunted growth in children less than five years, and inadequate intake of zinc in the diet. By design, this study focused on patients with CF with or without IR and IP. There is a need for large multicentric trials to improve the understanding of the nutritional status of zinc in these patients and to determine the amount needed and adequate zinc supplementation to improve the nutritional status with cystic fibrosis; we suggest conducting a multicentre international study and changing the approach to include the risk of zinc deficiency in health primary prevention.

## 5. Conclusions

Serum zinc was associated with nutritional status, expressed as BMI and weight-for-height Z-score. Moreover, dietary zinc intake was associated with nutritional status expressed as energy intake and weight-for-height Z-score. In the series, 23.5% (4/17) of patients with CF had inadequate dietary zinc intake and 17.6% (3/17) had hypozincemia. No patients with zinc deficient intake had hypozincemia. This situation of deficient dietary zinc intake without hypozincemia should alert us to a state of marginal deficiency of zinc. Therefore, we suggest that it should be considered that the percentage of patients with a deficient status of zinc could be much higher, and about 41% of our patients with CF would be at high risk of zinc deficiency and a zinc supplementation may be considered.

## Figures and Tables

**Table 1:** Baseline Characteristics of the Subjects (N = 17 *).

Characteristics	Mean ± SD or No. (%)	Median	Range
Female (%)	10 (58.8%)		
Male (%)	7 (41.2%)		
Age (years)	14.8 ± 8	15	2–31
Sick time (months)	159.7 ± 85	148	29–298
Genotype (%) (*n* − 17)			
Homozygous ∆F580	6 (35.3%)		
Heterozygous ∆F508	7 (41.2%)		
Other mutations	4 (23.5%)		
Management			
Lipase (IU/kg/day)	4881 ± 1469	5000	14,500–7200
Vitamin A (IU)	5500 ± 3438	5000	0–10,000
Vitamin E (IU)	223 ± 137	200	0–420
Vitamin D_3_ (IU)	578 ± 360	666	0–1333
Vitamin K (mg/w)	9.5 ± 4.3	10	0–20
Respiratory and Pancreatic Function			
Forced Vital Capacity (%)	83.9 ± 38.1	78.8	33–198
Forced Expired Volume in 1 s (%)	79.2 ± 25.8	78.9	29–129
Score Norman-Crispin	6.3 ± 5.5	6.5	0–19
Coefficient fat-absorption (%)	88.4 ± 8.6	91.3	72.3, 100
Nitrogen Balance	3.7 ± 5.3	2.8	−6.4–11.4
Anthropometric Assessment			
Body Mass Index (Kg/m^2^)	17.4 ± 2.6	16.3	14.2–23.4
Body Mass Index Z-score	−0.95 ± 1.1	−0.6	−3.8–0.6
Weight (kg)	37.9 ± 15.4	36.7	10.7–60.4
Height (cm)	143.9 ± 24.4	154.8	85–169.9
Weight-for-age Z-score	−1.2 ± 1.1	−1.3	−2.9–0.4
Height-for-age Z-score	−0.6 ± 1.1	−1.2	−1.9–0.92
Weight-for-height Z-score	0.22 ± 1.51	0.1	−1.4–4.58
Growth rate (cm/year)	5.12 ± 2.3	5.5	0.19–7.56
Blood Analytic			
Serum Zinc level (µg/dL)	87.2 ± 16.7	86	58–122
C-reactive protein (u/L)	4.6 ± 2.4	3.7	3.1–12
Erythrocyte sedimentation rate (mm/h)	13.1 ± 5	12	6–23
C-reactive protein (>4 u/L)	3 (17.7%)		
Erythrocyte sedimentation rate *	2 (11.8%)		
Prospective Dietary Survey			
Dietary zinc intake (%DRI)	97 ± 26.9	98.2	54.9–153.9
Zinc intake (mg/day)	14.5 ± 4	14.7	8.2–23.1
Energy intake (calories)	2594.9 ± 464.5	2672.4	1846–3409.6
Comorbidities (%)			
Undernutrition	5 (29.4%)		
Hypozincemia (µg/dL)	3 (17.6%)		
Dietary zinc deficiency (%DRI)	4 (23.5%)		
Respiratory insufficiency	9 (52.9%)		
Pancreatic insufficiency	13 (76.5%)		

* 17 cystic fibrosis patients were screened, included and analysed. No patients were excluded. Abbreviations: %DRI: Percentage of Dietary Reference Intake. * woman > 20 mm/h, men > 15 mm/h. IU: International Units. Vitamin D_3_: Cholecalciferol.

**Table 2:** Genotipe of fibrosis cystic patients.

Genotipe of Fibrosis Cystic Patients	Number
Delta F-508 homozygous	8
Delta F508/2183AA → G	2
Delta F-508 mutation in intrón 12 (1898 +1G → A)	1
Delta F-508/1717-IG-A	1
Delta F-508/S549R	1
Mutation 1717-1G	1
Mutation 1341-A → G exon 8	1
Mutation G → A exon 8, G673X exon 13	1
Mutation L97F exon 17	1
Total	17

**Table 3:** Differences between fibrosis cystic patients.

**Characteristics**	**Male**	**Female**	***p*** **-Value**
Age (years)	10.4 ± 7.2	17.2 ± 7.9	0.091
Serum zinc concentration (µg/dL)	93.3 ± 10.2	83 ± 19.5	0.223
Dietary zinc intake (mg/day)	12.8 ± 3.9	15.9 ± 3.8	0.142
Dietary zinc intake (%DRI)	85.6 ± 25.9	105.9 ± 25.5	0.140
**Colonisation**	**Yes**	**No**	***t*** **-value**
FVC	76.9 ± 24.2	94.5 ± 53.7	0.478
FEV_1_	74.9 ± 27.3	84.9 ± 27.5	0.511
**Respiratory function**	**Sufficient**	**Insufficient**	***t*** **-value**
Serum zinc concentration (µg/dL)	90 ± 16.6	84.8 ± 17.4	0.537
Dietary zinc intake (%DRI)	81.5 ± 20.8	112.5 ± 23.6	0.015
**Pancreatic function**	**Sufficient**	**Insufficient**	***t*** **-value**
Serum zinc concentration (µg/dL)	82 ± 1204	88.8 ± 17.9	0.417
Dietary zinc intake (%DRI)	103.2 ± 11.4	95.6 ± 29.5	0.484

FVC: forced vital capacity; FEV_1_: forced expired volume in 1 second; %DRI: Percentage of Dietary Reference Intake.
